# Effect of Selectively Etched Al-Rich and Si-Rich Microstructures on the Adhesion of Polyimide Coatings to SLM AlSi10Mg

**DOI:** 10.3390/ma19020385

**Published:** 2026-01-18

**Authors:** Jianzhu Li, Shuo Yang, Yujie Li

**Affiliations:** 1 State Key Laboratory of Advanced Welding and Joining, Harbin Institute of Technology at Weihai, Wenhua West Road 2, Weihai 264209, China; 2 School of Materials Science and Engineering, Harbin Institute of Technology at Weihai, Weihai 264209, China

**Keywords:** selective laser melting, AlSi10Mg, polyimide, surface microstructures, interfacial adhesion

## Abstract

Interfacial adhesion between selective laser-melted (SLM) AlSi10Mg and polyimide (PI) insulating coatings is often limited by mismatched physicochemical properties. To improve adhesion, Al-rich and Si-rich microstructured surfaces were fabricated on the XY plane (perpendicular to the build direction) and the Z plane (parallel to the build direction) by acidic and alkaline etching, exploiting the characteristic microstructure of SLM AlSi10Mg. Surface topography, chemical composition, and wettability were characterized, and interfacial mechanical performance was evaluated by shear and pull-off tests. The microstructures increased surface roughness and improved wettability. The shear strength rose from 2.6 ± 1.5 MPa for the polished surface to 43.2 ± 8.6 MPa. The polished surface showed a pull-off strength of 2.2 ± 0.25 MPa. In pull-off tests, failure mainly occurred within the dolly/adhesive/PI system, indicating that the interfacial tensile strength exceeded the strength of the adhesive system; the maximum measured pull-off strength was 29.0 ± 1.3 MPa. Fractography predominantly showed cohesive failure in PI on Al-rich microstructures. Si-rich microstructures exhibited mixed failure, including fracture of the Si skeleton and tearing of PI, together with interfacial microcracks.

## 1. Introduction

As electronic integration advances, structure–function integrated circuits are used in aerospace, precision instruments, and next-generation communication devices [1,2,3]. In this approach, conductive traces are built on the surface of structural housing. This reduces reliance on conventional printed circuit boards (PCBs). It can remove discrete PCBs and shorten circuit paths. It also combines electrical and mechanical functions within one structural unit. As a result, systems can be smaller and lighter, and mechanical motion is less likely to interfere with circuits [4,5,6]. Selective laser melting (SLM) is a metal additive manufacturing method. It can directly build AlSi10Mg components with complex shapes [7,8]. This provides a manufacturing route for structure–function integration.

Electrical insulation between circuits still requires a polymer insulating layer on the metal conductor. Polyimide (PI) is used in such coating systems because it has high dielectric strength, heat resistance, mechanical performance, and chemical stability [9]. However, differences in physicochemical properties and surface conditions between the metal substrate and the PI coating often limit interfacial bonding strength. Weak bonding can lead to interfacial voids and microcracks. It can also cause local blistering or delamination. These defects reduce coating integrity and can affect device stability during operation [10,11,12].

Two main strategies have been used to strengthen metal–polymer interfaces: geometric interlocking and surface chemical modification. Geometric interlocking is usually achieved by building macro- or micro-scale anchoring features on the surface, such as laser-ablated grooves, micropore arrays, or bioinspired “mushroom-like” structures. These features improve load transfer across the interface [13,14,15,16]. Surface chemical modification often uses adhesion promoters, such as silane coupling agents. These agents form chemical bonds within the interlayer and improve interfacial compatibility [17]. However, it remains difficult to make uniform interlocking features on complex three-dimensional surfaces. In addition, the stability of silane layers at elevated temperatures is uncertain. Their use under PI processing conditions, such as curing, also needs to be evaluated [18]. A more general surface treatment is therefore required for complex structures. Such a method should strengthen bonding while reducing process complexity.

For aluminum alloy components produced by selective laser melting (SLM), chemical etching is widely used as a pretreatment to remove oxide films and improve surface condition. Most studies, however, treat SLM alloys as compositionally uniform materials and apply non-selective roughening. They do not exploit the different reactions of the α-Al matrix and the eutectic Si phase. This limits controlled microstructure formation, guided by the underlying phases [19]. SLM AlSi10Mg consists of a cellular α-Al matrix and a continuous eutectic Si network [20]. The two phases etch at different rates in acidic and alkaline media. Phase-selective etching can therefore form Al-rich or Si-rich micro- and nanoscale features in situ [21,22]. These features provide a geometric basis for mechanical interlocking. Compared with random roughening, phase-selective structuring creates surface features with defined length scales. However, the links between surface features and interfacial strength remain unclear for acidic versus alkaline etching on different build planes. The related failure modes and their causes also need systematic analysis.

Here, a phase-selective chemical etching method is proposed to form Al-rich or Si-rich microstructures on SLM AlSi10Mg and to improve adhesion to a polyimide (PI) coating. The morphology, surface chemistry, and wettability resulting from acidic and alkaline etching on the XY and Z build planes are characterized. Interfacial strength is then quantified by shear and pull-off tests. Fractography is used to compare failure modes across different microstructures and to discuss the underlying causes. The results provide experimental evidence for the design and optimization of insulating coatings on additively manufactured metal components.

## 2. Materials and Methods

### 2.1. Materials and Specimen Preparation

The metal substrate was an AlSi10Mg alloy fabricated by selective laser melting (SLM). The feedstock was AlSi10Mg powder (5–45 µm; Bright Laser Technologies, Xi’an, China). The nominal powder composition (wt.%) was Si 9.80, Mg 0.40, Fe 0.15, Zn 0.03, Zr 0.01, Ti 0.01, with Al as the balance. SLM was carried out on an EOSINT M280 system under an argon (Ar) atmosphere. The oxygen level in the build chamber was kept below 50 ppm. The process parameters were as follows: laser power 370 W, scan speed 1300 mm s^−1^, hatch spacing 200 μm, and layer thickness 30 μm [23]. An alternating scan path was used. The scan direction was rotated by 67° between adjacent layers. To study the effect of build-plane orientation, the surface perpendicular to the build direction was defined as the XY plane. The side surface parallel to the build direction was defined as the Z plane.

The insulating material was a soluble polyimide (PI) precursor solution (20 wt.% solids; DuPont, Wilmington, DE, USA). The viscosity was >2000 mPa·s at 25 °C. The density was 1.05–1.12 g cm^−3^.

### 2.2. Surface Etching and PI Coating Preparation

After mechanical polishing, two types of surface microstructures were formed by acidic or alkaline etching. The acidic etchant was a HF/HNO_3_/H_2_O mixed solution (1:3:1 by volume). Samples were immersed at 25 °C for 7 s to obtain Al-rich microstructures (denoted as XY-Al and Z-Al). The alkaline etchant was an aqueous NaOH solution (60 g L^−1^). Samples were immersed at 25 °C for 90 s to obtain Si-rich microstructures (denoted as XY-Si and Z-Si). After etching, the samples were rinsed with deionized water, cleaned with anhydrous ethanol, and dried. They were then used for PI coating and characterization.

PI coatings were applied by brush. The PI precursor solution was brushed uniformly onto the treated and dried metal surface to form a wet film. The samples were then cured in a vacuum oven with stepwise heating. This gradually removed the solvent and completed imidization. The curing schedule was 100 °C for 0.5 h, 150 °C for 40 min, and 250 °C for 1 h. The heating rate was ~100 °C h^−1^ throughout. After curing, the samples were cooled in the oven to room temperature to obtain PI-coated specimens.

### 2.3. Characterization and Interfacial Adhesion Tests

Surface and interfacial morphologies were examined by scanning electron microscopy (SEM; MERLIN, Carl Zeiss, Oberkochen, Germany). Local elemental distributions were analyzed by energy-dispersive X-ray spectroscopy (EDS; Octane PLUS, EDAX, Mahwah, NJ, USA). Three-dimensional surface topography and roughness were measured by atomic force microscopy (AFM; Dimension XR, Bruker, Billerica, MA, USA). Surface chemical states were analyzed by X-ray photoelectron spectroscopy (XPS; ESCALAB, Thermo, Fisher Scientific, Waltham, MA, USA) using an Al Kα source. Survey and high-resolution spectra were collected, and binding energies were referenced to the C 1s peak at 284.8 eV. The Al 2p and Si 2p regions were fitted using constrained 2p spin–orbit doublets (ΔE = 0.44 eV for Al 2p and 0.605 eV for Si 2p; 2:1 area ratio). Wettability was measured using a contact angle goniometer (SZ-CAMC33, Shanghai Sunzern Instrument Co., Ltd., Shanghai, China) with the static sessile drop method. The droplet volume was set to 3 µL, and the dispensing speed was 1 µL/s. Deionized water and diiodomethane were used as probe liquids. Each sample was measured at least three times at different locations, and the results were averaged. The surface free energy, including its dispersive and polar components, was calculated using the Owens–Wendt–Rabel–Kaelble (OWRK) model [24].

**Figure 1:**
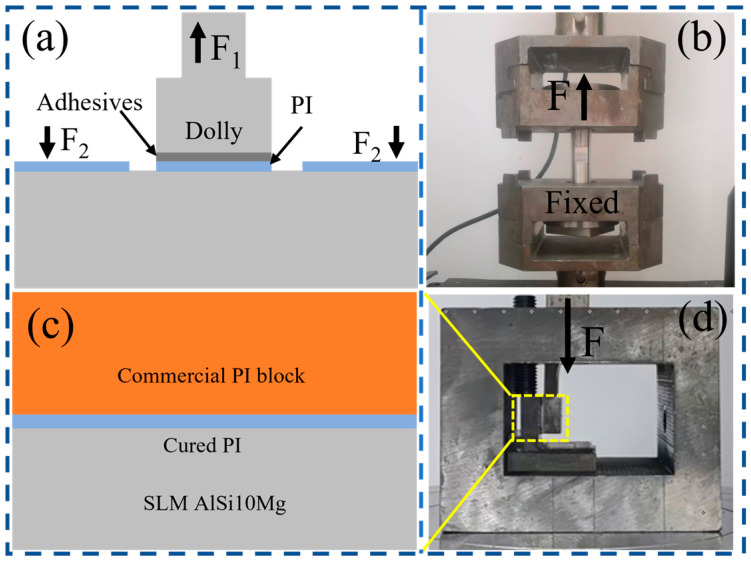
Schematics and experimental setups for adhesion tests: (**a**,**b**) pull-off test configuration and fixture; (**c**,**d**) compression-loaded shear test configuration and fixture.

Interfacial adhesion was evaluated by pull-off and compression-loaded shear tests. All mechanical tests were carried out at room temperature. Each condition included five valid repeats (*N* = 5) to ensure statistical reliability. Pull-off tests followed ISO 4624:2023 [25]. First, aluminum alloy dollies (20 mm in diameter) were bonded to the cured PI surface using a two-part epoxy adhesive. The adhesive was cured for 24 h at room temperature. The specimens were loaded at 1 mm min^−1^ along the dolly axis (perpendicular to the coating surface) until failure. The maximum load was recorded and used to calculate the adhesion strength. A schematic of the setup is shown in Figure 1a,b.

Shear tests used a compression-loaded configuration. PI coatings were prepared and cured on treated SLM AlSi10Mg coupons (10 mm × 10 mm × 8 mm). A commercial PI block of the same size was then bonded to the PI coating using a PI-specific adhesive. This formed a shear joint with an overlap area of 10 mm × 10 mm. The adhesive was cured for 24 h at room temperature. The shear loading direction was parallel to the bonded interface. The joint was loaded at 1 mm min^−1^ until failure. The shear strength was calculated as the maximum load divided by the initial overlap area (10 mm × 10 mm). A schematic of the setup is shown in Figure 1c,d.

## 3. Results and Discussion

### 3.1. Surface Features

The SLM AlSi10Mg substrate consists of a cellular α-Al matrix and a Si-rich phase. In acidic solutions, the Si-rich phase etches faster [26]. After etching, a residual α-Al framework remains on the surface. This produces an Al-rich microstructure. By contrast, in alkaline solutions, α-Al etches faster. The Si-rich framework is retained [27], resulting in a Si-rich microstructure.

**Figure 2:**
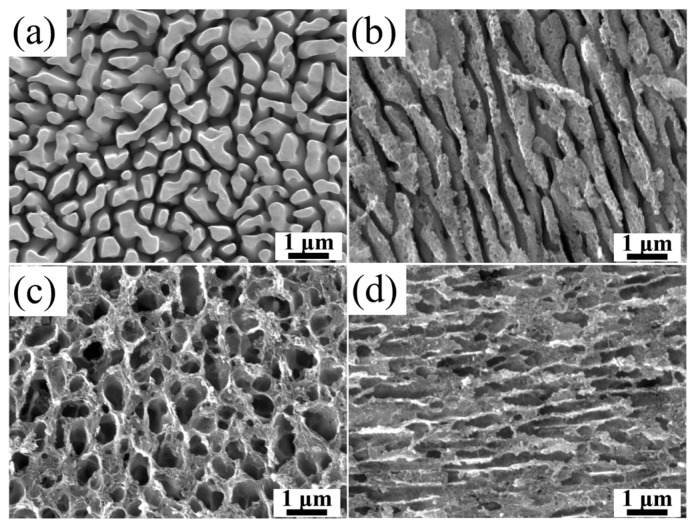
Representative SEM images of Al-rich and Si-rich surface microstructures onSLM AlSi10Mg obtained under the selected etching conditions: (**a**) XY-Al (acidic etching, 7 s), (**b**) Z-Al (acidic etching, 7 s), (**c**) XY-Si (alkaline etching, 90 s), and (**d**) Z-Si (alkaline etching, 90 s).

After 7 s of acidic etching, the XY plane showed an aluminum-island structure (denoted XY-Al; Figure 2a). The islands had an equivalent diameter of ~0.35 μm. The island spacing was ~0.20 μm. Deep valley-like gaps formed between islands. Bridge-like connections were also observed locally. These bridges provide channels for infiltration of the polymer precursor. This may contribute to geometric interlocking after curing. Under the same acidic condition (7 s), the Z plane developed an Al-rich stripe structure aligned with the build direction (denoted Z-Al; Figure 2b). The structure mainly consisted of continuous Al columns. The characteristic width *w* was ~0.38 μm.

After 90 s of alkaline etching, the XY plane formed a porous Si network (denoted XY-Si; Figure 2c). The network showed a mesh-like pore structure. The pore walls and skeleton boundaries were distinct. Locally, through-pores were observed. Under the same alkaline condition (90 s), the Z plane formed a Si tubular structure oriented along the Z direction (denoted Z-Si; Figure 2d). The characteristic width was ~0.25 μm. The local merging of adjacent channels was observed.

**Figure 3:**
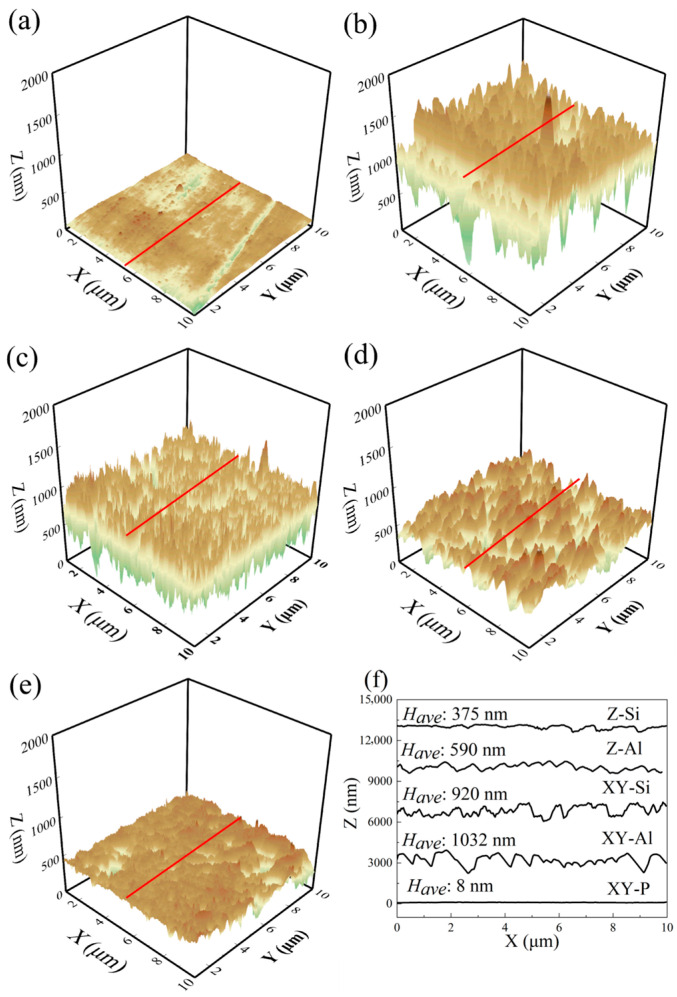
AFM 3D topographies and line profiles of samples with different surface states. (**a**) XY-P; (**b**) XY-Al; (**c**) XY-Si; (**d**) Z-Al; (**e**) Z-Si; (**f**) corresponding line-scan height profiles (profiles are vertically offset for clarity, The red lines indicate the locations of the line scans shown in (**f**). The colors represent the surface height).

Atomic force microscopy (AFM) was used to measure three-dimensional surface topography. The areal roughness parameters, including the arithmetical mean height (*S_a_*), root-mean-square height (*S_q_*), and developed interfacial area ratio (*Sdr*), were calculated according to ISO 25178 [28]. To quantify the interlocking depth, we introduced *H_ave_*, defined as the arithmetic mean of the vertical height differences between adjacent peaks and valleys ($H_{ave}=\frac{1}{n}\sum (Z_{peak}-Z_{valley})$). Unlike the global roughness Sa, *H_ave_* specifically measures the average amplitude of local micro-structures available for anchoring. Representative AFM 3D maps and 2D profiles are shown in Figure 3. The roughness parameters are summarized in Table 1.

As shown in Table 1, the polished surface (XY-P) was smooth, with *S_a_* = 9.7 nm and *Sdr* = 0.6%. After chemical etching, *S_a_*, *S_q_*, *H*_ave_, and *Sdr* all increased for the four microstructured surfaces. The overall roughness followed the trend XY-Al > XY-Si > Z-Al > Z-Si > XY-P. For the extreme cases, XY-Al showed the highest values, with *S_a_* = 263.2 nm and *Sdr* = 110.6%. Z-Si showed the lowest values among the microstructured samples, with *S_a_* = 138.3 nm and *Sdr* = 49.7%. Figure 3 also shows that the XY-plane samples had larger height variations and a higher area increase than the Z-plane samples.

An increase in *Sdr* indicates a larger geometric surface area after etching. This may increase the effective contact area and may contribute to geometric interlocking [29]. It should be noted that AFM measurements are affected by the tip radius and sidewall effects. The actual depth of narrow pores or grooves can be underestimated [30]. However, the infiltration depths confirmed by the subsequent cross-sectional analysis (see Section 3.3) were consistent with the topographic trends, validating the geometric characterization. Therefore, *S_a_* and *S_q_* were used as the primary metrics to compare relative height variations among samples.

### 3.2. Wettability and Surface Energy

**Figure 4:**
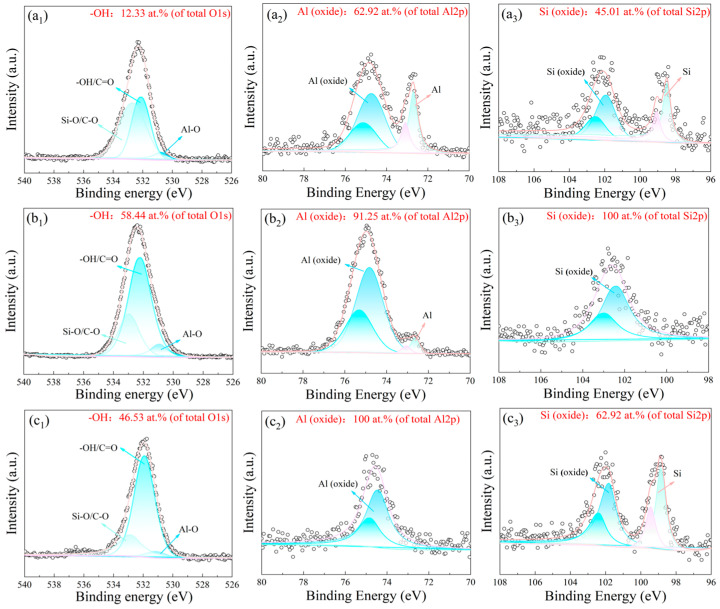
High-resolution XPS spectra of SLM AlSi10Mg surfaces with different surface states: polished (XY-P, (**a_1_**–**a_3_**)), Al-rich microstructure (XY-Al, (**b_1_**–**b_3_**)), and Si-rich microstructure (XY-Si, (**c_1_**–**c_3_**)). For each surface, the deconvoluted regions include O 1s, Al 2p, and Si 2p. The Al 2p and Si 2p envelopes were fitted using constrained 2p3/2 and 2p1/2 doublets (fixed splitting, 2:1 area ratio).

Figure 4 shows high-resolution XPS spectra of the polished reference (XY-P) and two representative etched surfaces (XY-Al and XY-Si). The O 1s, Al 2p, and Si 2p regions are included. Peak fitting was used to separate and correct the overlapped C–O and C=O contributions in the O 1s region. The contribution of oxygenated carbon species to O 1s was assessed using the C 1s spectra (Figure S1). Accordingly, the reported –OH fraction represents an apparent hydroxyl-related contribution after considering adventitious carbon effects.

The O 1s envelope was resolved into three components: Al–O at ~531.0 eV, an apparent –OH-related component at 531.5–532.0 eV (overlapping with possible C=O contributions), and a Si–O-related component at 532.8–533.3 eV (overlapping with possible C–O contributions). Compared with XY-P, the etched samples showed an apparent increase in the relative –OH content. The –OH fraction was 12.33% for XY-P. It increased to 58.44% for XY-Al and 46.53% for XY-Si. These results indicate that chemical etching increased the surface –OH content and, in turn, increased the number of polar sites.

The fitted Al 2p and Si 2p peaks were used to describe surface chemical states. In the Al 2p region, Al^0^ was located at 72.6–72.8 eV and Al^3+^ at 74.4–74.7 eV. In the Si 2p region, Si^0^ was located at 99.3–99.5 eV and Si^4+^ at 101.0–103.5 eV. On XY-P, Al^3+^ accounted for 62.92% and Si^4+^ for 45.01%. On XY-Al, Al^3+^ accounted for 91.25%, and Si was mainly in the oxidized state (close to 100%). On XY-Si, Al^3+^ was 100% and Si^4+^ was 62.92%. These changes indicate the formation of an oxide-rich surface layer after etching, which is consistent with the increased –OH fraction derived from O 1s fitting.

**Figure 5:**
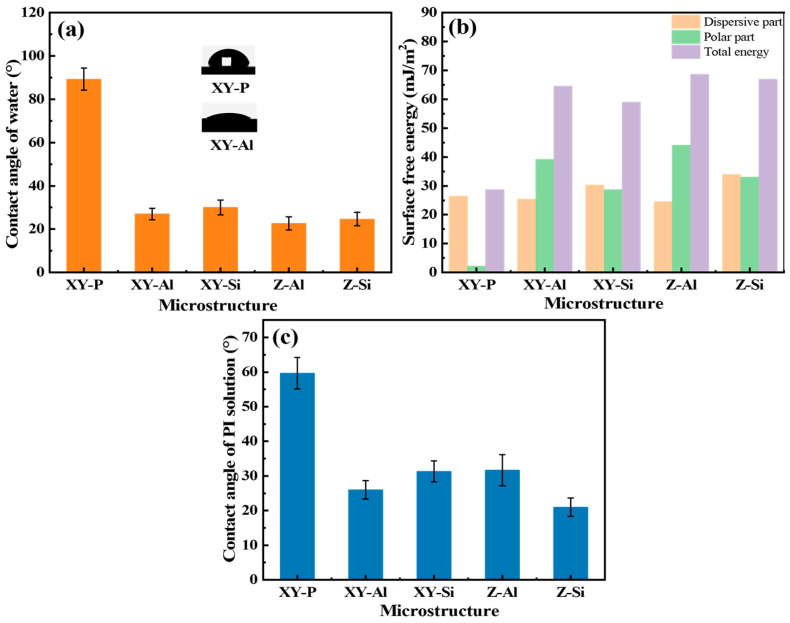
Wettability and surface free energy of polished and etched SLM AlSi10Mg surfaces: (**a**) water contact angle, (**b**) surface free energy and its dispersive/polar components calculated using the OWRK method, and (**c**) contact angle of the PI precursor solution on XY-P, XY-Al, XY-Si, Z-Al, and Z-Si surfaces.

Figure 5 compares wettability and surface free energy for the polished surface (XY-P) and the etched surfaces. The deionized-water contact angle was ~89° on XY-P. It decreased to 25–32° after etching. A lower contact angle is associated with higher roughness and greater surface polarity. AFM showed that etching increased roughness and, consequently, the geometric surface area. XPS showed a higher fraction of polar species, including –OH groups. Therefore, the combined changes in roughness and surface polarity were associated with improved wettability. The surface free energy followed the same trend. Compared with XY-P, the total surface energy increased from ~30 mJ m^−2^ to 60–75 mJ m^−2^ for the etched surfaces. The increase was mainly due to the polar component. This agrees with the higher –OH fraction observed by XPS.

To assess coating processability, the contact angle of the PI precursor solution was measured on each surface. The contact angle was 60–65° on XY-P. It decreased to 20–35° on the etched surfaces. A lower contact angle and higher surface energy are expected to facilitate spreading of the PI precursor and infiltration into microstructural gaps. This can increase the actual contact area between the coating and the substrate [31].

### 3.3. Adhesion and Failure

**Figure 6:**
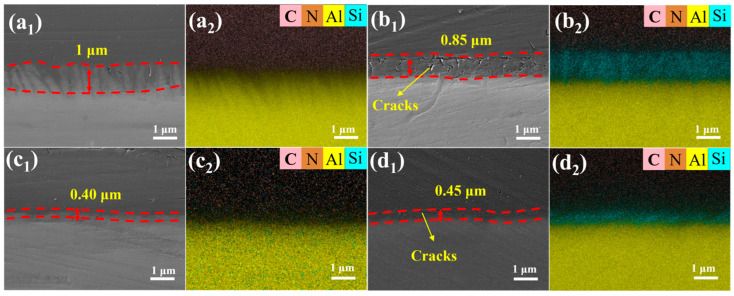
Cross-sectional SEM images and corresponding EDS elemental maps of AlSi10Mg/PI interfaces on microstructured SLM AlSi10Mg surfaces: (**a_1_**,**a_2_**) XY-Al, (**b_1_**,**b_2_**) XY-Si, (**c_1_**,**c_2_**) Z-Al, and (**d_1_**,**d_2_**) Z-Si. The red dashed lines outline the infiltration region, and the red double arrows indicate the infiltration depth.

Figure 6 shows cross-sectional morphologies and EDS elemental maps for the AlSi10Mg/PI interface on the four etched surfaces (XY-Al, XY-Si, Z-Al, and Z-Si). In all etched samples, the PI coating formed a continuous layer on the substrate. By contrast, the polished reference (XY-P) showed coating–substrate separation during cross-section preparation (Figure S2), suggesting that the interface was sensitive to sample-preparation disturbances.

On the XY build plane, the PI coating on XY-Al filled the surface valleys well. The average infiltration depth was ~1.00 μm (Figure 6(a_1_,a_2_)). The interface contour was continuous. No through-cracks or noticeable pores were observed. For XY-Si, the average infiltration depth was ~0.85 μm (Figure 6(b_1_,b_2_)). Scattered microcracks were observed near the interface. The EDS maps showed that the C and N signals were mainly located in the PI layer, whereas the Al and Si signals primarily came from the alloy substrate. For the Si-rich samples (XY-Si and Z-Si), the Si signal was concentrated in the skeleton regions. The C and N signals were enhanced in the gaps. This indicates that the PI precursor entered the gaps within the Si skeleton and remained there after curing. On the Z build plane, the average infiltration depth was ~0.40 μm for Z-Al (Figure 6(c_1_,c_2_)). The interface was essentially continuous, with no apparent cracks. The average infiltration depth was ~0.45 μm for Z-Si (Figure 6(d_1_,d_2_)). Scattered microcracks were also observed near the interface. The EDS distribution was similar to that of XY-Si, with Si concentrated in the skeleton regions and C and N enhanced in the gaps.

Microcracks observed near the XY–Si and Z–Si interfaces may be associated with residual tensile stresses that develop during cooling after PI curing. Residual stress was not directly measured in the present work; therefore, thermal-expansion mismatch is discussed as one plausible contributing factor. Using representative literature values, the CTE of Si is ~3.4–3.6 × 10^−6^ K^−1^ at 200–300 °C [32], whereas PI films can exhibit CTE values on the order of ~18.8 × 10^−6^ K^−1^ over 50–250 °C [33]; SLM AlSi10Mg typically shows CTE values of ~23–26 × 10^−6^ K^−1^ around 250 °C [34]. The resulting thermal strain mismatch on cooling from 250 °C to 25 °C is estimated as Δε ≈ (α_PI_ − α_Si_)ΔT ≈ 3.4 × 10^−3^ for PI/Si, which is larger than that for PI/AlSi10Mg (Δε ≈ (α_AlSi10Mg_ − α_PI_)ΔT ≈ 1.3 × 10^−3^). Such mismatch may concentrate local stresses at rigid Si-rich skeleton features and facilitate crack initiation at the observed length scales. In addition, microcracks could also arise from etching-induced stress concentration, local fragility of the Si skeleton, and shrinkage during PI curing.

**Figure 7:**
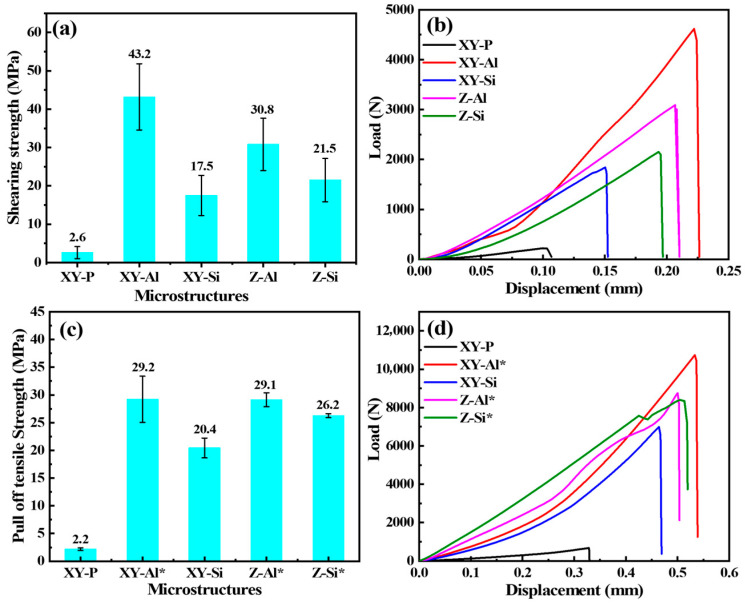
Interfacial adhesion of AlSi10Mg/PI under shear and pull-off loading: (**a**) interfacial shear strength, (**b**) representative load–displacement curves in shear, (**c**) pull-off tensile strength (interfacial tensile strength), and (**d**) representative load–displacement curves in pull-off. The asterisk (*) indicates that pull-off failure occurred within the dolly/adhesive/PI system (adhesive failure).

Figure 7a,b show the shear strength of the PI/AlSi10Mg interface and representative load–displacement curves for different surface states. As shown in Figure 7a, the polished surface (XY-P) had a shear strength of 2.6 ± 1.5 MPa. After etching to form microstructures, the shear strength increased to 17.5–43.2 MPa. The values were 43.2 ± 8.6 MPa for XY-Al, 30.8 ± 6.8 MPa for Z-Al, 21.5 ± 5.6 MPa for Z-Si, and 17.5 ± 5.2 MPa for XY-Si. The trend was XY-Al > Z-Al > Z-Si > XY-Si > XY-P. On the same build plane, Al-rich microstructures showed higher shear strength than Si-rich microstructures. For the same microstructure type, the XY plane showed higher shear strength than the Z plane.

Figure 7b shows an approximately linear response at the initial loading stage for all samples. The initial tangent slope can be used to describe interfacial shear stiffness. Compared with XY-P, the microstructured samples showed a larger initial slope. After the peak load, the curves dropped rapidly. This behavior is consistent with mainly brittle failure.

**Figure 8:**
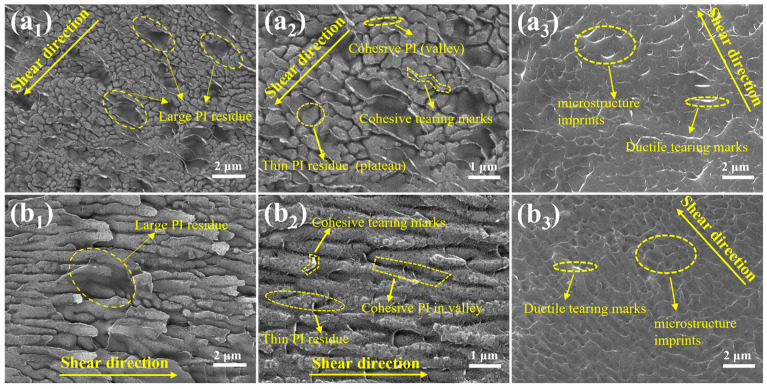
Shear-fracture morphologies of Al-rich microstructured AlSi10Mg/PI interfaces: XY-Al (**a_1_**–**a_3_**) and Z-Al (**b_1_**–**b_3_**). The images are grouped by failure location: (**a_1_**,**a_2_**,**b_1_**,**b_2_**) show the fracture surfaces on the AlSi10Mg substrate side at low and high magnifications; (**a_3_**,**b_3_**) show the fracture surfaces on the PI coating side; arrows indicate the shear direction.

Figure 8 shows fracture surfaces after shear failure for the two Al-rich microstructures (XY-Al and Z-Al). The two samples showed similar features. On the substrate side, continuous PI residues and tearing marks were observed. On the PI side, imprint features complementary to the substrate microstructures were preserved, together with tear stripes. These features are consistent with polymer penetration into the microstructure and geometric interlocking at the interface of Al-rich microstructures. Cracks mainly propagated within the PI coating. The dominant failure mode was cohesive failure.

Although cohesive failure dominated for both XY-Al and Z-Al, their shear strengths differed. This difference correlates with the interlocking length scale inferred from the PI infiltration depth and roughness/area parameters. For XY-Al, PI infiltrated deeper into surface valleys (~1.00 μm). The height variation and the increase in area were also larger. These geometric factors may increase the interlocked volume and may increase crack-path tortuosity, which is expected to raise the energy required for shear fracture. By contrast, PI infiltration was shallower for Z-Al (~0.40 μm), suggesting a smaller interlocking length scale and a lower measured shear strength.

**Figure 9:**
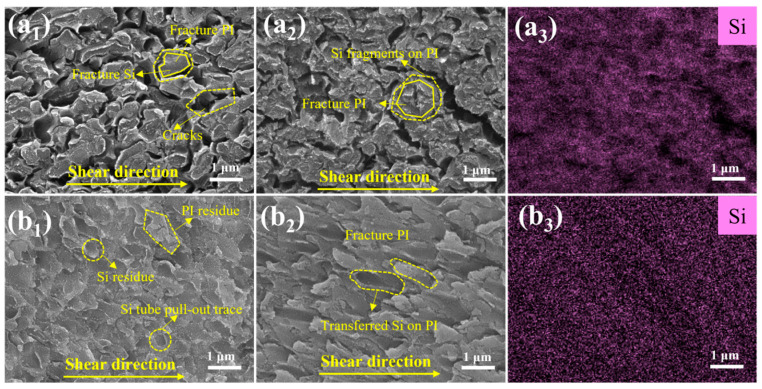
Shear-fracture morphologies and Si elemental maps of Si-rich microstructured AlSi10Mg/PI interfaces: XY-Si (**a_1_**–**a_3_**) and Z-Si (**b_1_**–**b_3_**). The panels display: (**a_1_**,**b_1_**) the AlSi10Mg substrate side; (**a_2_**,**b_2_**) the PI coating side; and (**a_3_**,**b_3_**) the corresponding Si elemental maps; arrows indicate the shear direction.

Figure 9(a_1_–a_3_) shows fracture surfaces and Si elemental maps for the XY-Si sample after shear failure. On the substrate side, through-cracks and step-like fracture features were observed. This suggests brittle fracture of the Si-rich skeleton under shear loading. The brittle features may be related to thermal-expansion mismatch between PI and Si. During cooling after curing, the mismatch may generate local tensile residual stress near the interface. This may facilitate crack initiation in the Si-skeleton region. During subsequent shear loading, fracture of the Si skeleton was observed, together with tearing of the PI coating. Some Si fragments were transferred to the PI side. On the PI side, tearing marks and attached Si fragments were observed. The Si map showed a continuous strong signal in the same region. This is consistent with the transfer of Si fragments from the substrate side to the PI side during shear.

Figure 9(b_1_–b_3_) shows fracture surfaces and Si elemental maps for the Z-Si sample after shear failure. On the substrate side, PI residues and Si-rich regions were observed. Stripe-like pull-out grooves were also present. These features suggest fracture of the Si skeleton during shear, with local skeleton pull-out. On the PI side, continuous tearing ridges were observed. The Si signal was enhanced in the corresponding region. This also supports the transfer of Si fragments to the PI side. Overall, the Si-rich samples showed mixed shear failure. Brittle failure of the Si skeleton appeared first. It was accompanied by tearing ridges in the PI coating and transfer of Si fragments.

It should be noted that the etching treatments modify surface topography and surface chemistry simultaneously. Therefore, the geometric factors (e.g., Sa, Sdr, and PI infiltration depth) and the chemical/energetic factors (e.g., XPS-indicated oxide/hydroxyl features and surface free energy) cannot be independently varied in the present design. Accordingly, the present results are discussed in terms of empirical correlations between the measured surface descriptors and the observed adhesion and failure features, rather than as definitive evidence of causality.

**Figure 10:**
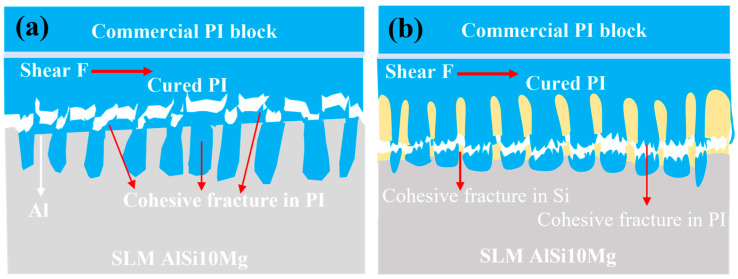
Schematic of shear-failure modes for (**a**) Al-rich (XY-Al) microstructured interfaces and (**b**) Si-rich (XY-Si) microstructured interfaces.

Figure 10 summarizes the dominant shear-failure features for two representative microstructured interfaces: the Al-rich interface (XY-Al) and the Si-rich interface (XY-Si). For XY-Al, continuous PI residues on the substrate side and complementary imprints on the PI side indicate that shear fracture occurred mainly within the PI coating. This behavior is consistent with an interface strengthened by polymer infiltration into the Al-rich microstructure, together with the improved wetting observed after etching. For XY-Si, through-cracks and step-like features on the substrate side, together with Si transfer signals on the PI side, suggest that fracture of the Si-rich skeleton and PI tearing both contributed to shear failure. Thermal-expansion mismatch during cooling may also contribute to local stress concentration and crack initiation near Si-rich regions. Overall, the measured shear-strength difference between XY-Al and XY-Si is associated with different interfacial architectures and failure features. The present results provide empirical correlations rather than definitive mechanistic proof.

Figure 7c,d show the normal tensile strength from the pull-off test and representative load–displacement curves for different surface states. The polished surface (XY-P) showed a pull-off strength of 2.2 MPa. After surface microstructuring, the pull-off strength increased. XY-Si reached 20.4 MPa. It should be noted that XY-Al, Z-Al, and Z-Si mainly failed cohesively within the dolly/adhesive/PI system during testing. Therefore, the measured values (~29.0 MPa) reflect the limit of the testing adhesive rather than the interface itself. These results should be interpreted as a conservative lower bound, implying that the true interfacial normal tensile strength exceeds 29.0 MPa. This indicates that the normal load capacity of these microstructured interfaces exceeded that of the adhesive system used in this test. Figure 7d shows that the polished sample reached a peak at a low load and failed rapidly. The microstructured samples showed higher peak loads and larger displacements. For XY-Al, Z-Al, and Z-Si, the peak corresponded to cohesive failure within the dolly/adhesive/PI system.

**Figure 11:**
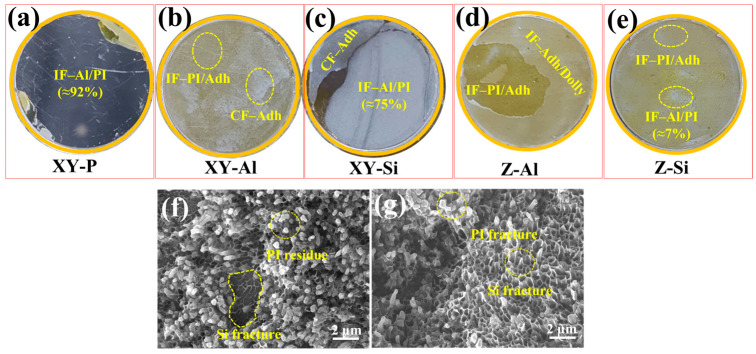
Pull-off fracture appearances of AlSi10Mg/PI interfaces: dolly-side macroscopic failure modes for XY-P, XY-Al, XY-Si, Z-Al, and Z-Si (**a**–**e**), and SEM fractography of XY-Si on the AlSi10Mg side (**f**) and PI side (**g**). Failure modes are denoted as IF–Al/PI (interfacial failure at the AlSi10Mg/PI interface), IF–PI/Adh (interfacial failure at the PI/adhesive interface), CF–Adh (cohesive failure within the adhesive layer), and IF–Adh/Dolly (interfacial failure at the adhesive/dolly interface); IF and CF denote interfacial failure and cohesive failure, respectively.

Figure 11a–e shows macroscopic fracture surfaces on the dolly side after the pull-off test. They were used to identify the failure location and type. The polished sample (XY-P; Figure 11a) showed interfacial failure at the Al/PI interface (IF-Al/PI) in ~92% of the area. This indicates that debonding at the interface was more likely under normal tension. For the microstructured samples (XY-Al, Z-Al, and Z-Si; Figure 11b,d,e), the fracture surfaces were mainly characterized by interfacial failure at the PI/adhesive interface (IF-PI/Adh) and cohesive failure within the adhesive (CF-Adh). This indicates that the adhesive’s strength limited their normal tensile strength. In contrast, the Si-rich XY-Si sample (Figure 11c) still showed a high fraction of IF-Al/PI (~75%). This suggests that, under normal tension, this interface tended to fail in a mode similar to that of the Al/PI interface.

To further clarify the failure mechanism of XY-Si, Figure 11f,g show microscopic features of its fracture surfaces. On the substrate side, PI residues and fracture features of the Si-rich skeleton were observed. On the PI side, tearing ridges and tearing marks were observed, together with fracture traces of the Si phase. These results indicate that Si-skeleton fracture and PI damage occurred simultaneously during pull-off, resulting in a mixed fracture mode. This is consistent with the high IF-Al/PI fraction observed in the macroscopic analysis.

The practical effectiveness of phase-selective etching is anticipated to depend on specific etching conditions and the local microstructure of the SLM material. Variations in parameters such as etching duration, solution concentration, temperature, and agitation can alter the degree of phase-selective dissolution. These changes subsequently influence the characteristic height scale and connectivity of the generated surface features, which are critical for polymer infiltration and adhesion. Furthermore, the SLM process inherently introduces microstructural heterogeneity, including melt-pool boundaries and variations in Si-network continuity, that can lead to a spatially non-uniform etching response. For complex three-dimensional components, etching uniformity may be further compromised by mass-transport limitations, bubble entrapment, and local shadowing within recessed regions. Therefore, robust practical implementation requires the definition of a stable processing window and verification of reproducibility across representative locations and geometries.

## 4. Conclusions

This study shows that selective chemical etching, guided by microstructural differences in SLM AlSi10Mg, can create Al-rich and Si-rich surface features and can improve the adhesion strength of a polyimide (PI) coating under the investigated etching conditions. The main conclusions are as follows:

(1) After etching, surface morphology and wettability improved. The interfacial shear strength increased from 2.6 ± 1.5 MPa for the polished surface to a maximum of 43.2 ± 8.6 MPa.
(2) Fractography and cross-sectional observations indicate predominantly cohesive fracture at Al-rich interfaces. Si-rich interfaces show mixed failure, with fracture of the Si skeleton and PI tearing. Microcracks were also observed near the interface. Thermal-expansion mismatch between PI and Si may contribute to microcrack formation at Si-rich interfaces.
(3) In pull-off tests, the peak load for XY-Al, Z-Al, and Z-Si was limited by cohesive failure within the dolly/adhesive/PI system. The maximum measured pull-off strength was 29.0 ± 1.3 MPa. Failure occurred mainly within the dolly/adhesive/PI system rather than at the Al/PI interface. Therefore, future work should employ a higher-strength adhesive system or an alternative tensile test configuration to quantify the interfacial tensile strength. In addition, interfacial durability should be evaluated under thermal cycling and environmental exposure, with particular attention paid to microcrack formation at Si-rich interfaces.

## Figures and Tables

**Table 1:** Roughness and surface area parameters (*S_a_*, *S_q_*, *H_ave_*, *Sdr*) of samples with different surface states.

Sample	*S_a_* (nm)	*S_q_* (nm)	*H_ave_* (nm)	*Sdr* (%)
XY-P	9.7	12.9	8	0.6
XY-Al	263.2	332.7	1032	110.6
XY-Si	234.8	276.6	920	92.5
Z-Al	181.2	226.6	590	78.3
Z-Si	138.3	141.1	375	49.7

## Data Availability

The original contributions presented in this study are included in the article/Supplementary Materials. Further inquiries can be directed to the corresponding author.

## Supplementary Materials

The following supporting information can be downloaded at: https://www.mdpi.com/article/10.3390/ma19020385/s1. Figure S1: High-resolution C1s XPS spectra of SLM AlSi10Mg surfaces with different surface states; Figure S2: Cross-sectional SEM image of the polished (XY-P) AlSi10Mg/PI interface showing an interfacial gap introduced during metallographic preparation.
