# Supplementary material for: Effect of Selectively Etched Al-Rich and Si-Rich Microstructures on the Adhesion of Polyimide Coatings to SLM AlSi10Mg
Source: Materials (Basel). 2026 Jan 18;19(2):385. doi: 10.3390/ma19020385 (PMC12842816; doi:10.3390/ma19020385)
Supplement: Supplementary file 1 [file materials-19-00385-s001.zip › materials-4101022-supplementary.pdf]

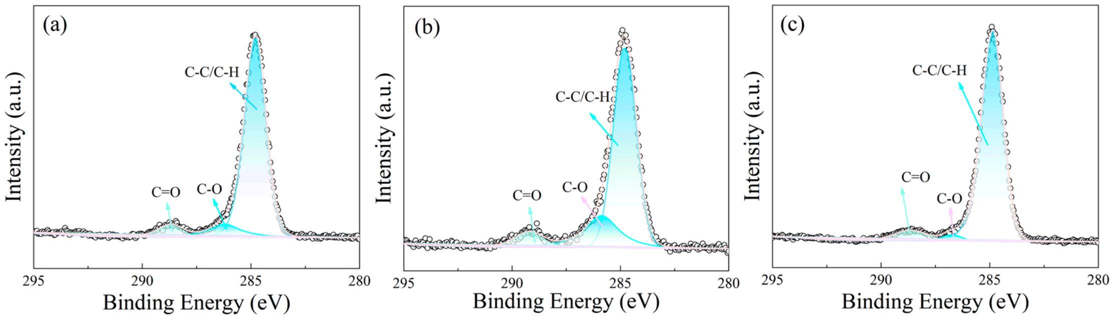

Figure S1. High-resolution C1s XPS spectra of SLM AlSi10Mg surfaces with different surface states: polished (XY-P, a), Al-rich microstructure (XY-Al, b), and Si-rich microstructure (XY-Si, c). The deconvoluted components are assigned to C–C/C–H, C–O, and C=O.

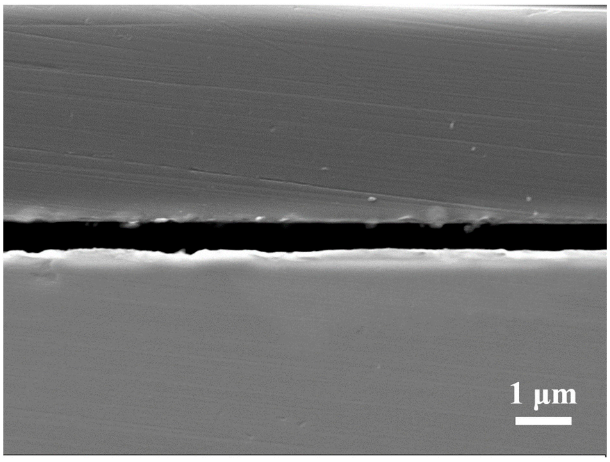

Figure S2. Cross-sectional SEM image of the polished (XY-P) AlSi10Mg/PI interface showing an interfacial gap introduced during metallographic preparation.
